# Universal Transfers, Tax Breaks and Fertility: Evidence from a Regional Reform in Norway

**DOI:** 10.1007/s11113-023-09793-z

**Published:** 2023-05-23

**Authors:** Rannveig Kaldager Hart, Taryn A. Galloway

**Affiliations:** 1 Centre for Fertility and Health, Norwegian Institute of Public Health, Oslo, Norway; 2 Research Department, Statistics Norway, Oslo, Norway

**Keywords:** Fertility, Cash transfers, Quasi experiment, Difference-in-difference, Family policy

## Abstract

The relatively high fertility in the Nordic countries has attracted considerable academic and political interest. Still, the causal relationship between economic circumstances and fertility in the Nordic context is poorly understood. This paper estimates the effect of tax breaks and universal transfers on fertility in the Nordic context. We analyze the fertility effects of a regional child benefit and tax reform implemented in the northern municipalities of the Norwegian county Troms, using the southern municipalities of the same county as a plausible and empirically similar control group. We use a difference-in-difference/event study design, and estimate multivariate models on individual-level data from administrative registers for the full population. The reform increased fertility among women in their early 20 s. The effects are concentrated among unmarried women, who received the largest subsidies. Our findings suggest that favorable economic conditions have contributed to the relatively high fertility in the Nordic countries.

**Supplementary Information:**

The online version contains supplementary material available at 10.1007/s11113-023-09793-z.

## Introduction

About a third of the world’s children, and nearly 90 percent of children in Europe and Central Asia, receive some sort of unconditional cash transfer (ODI/UNICEF, 2020). Unconditional transfers to families with children reduce social inequality and child poverty, without the potential stigma and added bureaucracy conditional benefits may entail (ibid). Because transfers reduce the direct cost of cost of raising children, they may increase birth rates. Pro-natalistic effects have also been among the motivations for recent expansion and introductions of universal child transfers (González, 2013; Milligan, 2005).

Over the last decade, Europe and the US have experienced a sharp fertility decline (Matysiak et al., 2020), and the proportion of European countries with polices aimed at increasing fertility has risen from 51 percent in 2005 to 67 percent in 2015 (United Nations, 2018). Public support to families goes together with relatively high fertility in Western countries (Kalwij, 2010). Still, isolating the effect of each component has proven difficult, as extensive family policies go together with other factors with that facilitate childbearing, such as economic growth, a family friendly culture and a labor market allowing good work-family balance (Gupta et al., 2008). The COVID-19 pandemic has given rise to both a severe economic downturn and indications of (further) falling fertility (Aassve et al., 2020). This makes efficient policy making, based on a precise understanding of the fertility effects of each policy component, more crucial than ever.

A small number of previous studies have estimated the effect of universal transfers and tax breaks on fertility in a credibly causal design (see Bergsvik et al. (2021) for an overview). Overall the results suggest small positive, yet transitory, effects. Exogenous variation is obtained through various reform designs, including increased transfers in the Canadian province Quebec (Ang, 2015; Milligan, 2005), a German reform that affected high and low earners differently (Riphahn & Wiynck, 2017) and the Spanish baby bonus (González, 2013). However, the evidence base is thin and based on a small number of contexts, with variation in benefits due to reforms often found at higher parities.

To the best of our knowledge, no previous study has estimated the effect of universal transfers and tax breaks on fertility in the Nordic context. The family policies in the Nordic welfare states reduce the direct cost of children (Esping-Andersen, 2013; Rønsen & Skrede, 2010). The substantial fertility fall in the Nordic countries after the 2009 recession hints at the importance of economic factors (Comolli et al., 2021). Andersen et al. (2018) finds some evidence of a postponement effect of increased cash-for-care (CFC) transfers in Norway. However, cash-for-care is conditional on not using publicly subsidized childcare, and changes could impact fertility indirectly by causing a longer career break after birth for mothers.

In this paper, we analyze a regional reform in Norway in a quasi-experimental design. In 1989–90, transfers to families with children increased in Northern Troms (intervention region), with no change in the comparable and bordering region Southern Troms (control region). We compare the fertility development for women in the two regions using difference-in-difference/event study designs. Our analysis is based on detailed, high quality data on fertility, education, income and marital status for the full female population in this region, drawn from administrative registers. To avoid bias from selective migration, place of residence and all potentially endogenous covariates are measured prior to the reform.

When fully implemented, the reform increased the universal child allowance for children aged 0–18 years by about 3600 NOK yearly, about 575 1990-USD.^1^ Regional tax breaks improved of the economic situation of women in the treatment region relative to the control region further. Tax breaks were largest for single mothers, meaning that we might expect the strongest effects on nonmarital fertility. It also follows that the reform may act as a disincentive to marriage, a hypothesis we test empirically. In contrast to previous studies, the nature of the reform allows for testing effects on maternal age at first birth, potentially more malleable by policy changes than number of children (Gauthier, 2007).

Results show that the reform increased fertility among women in their early 20 s. The effect is most marked for first births, but there are also tendencies of increases in second and third birth. This finding is consistent across specifications and robustness tests. We find the strongest response in the age group where earned income is lowest, and the relative economic improvement due to the reform largest. There is also an increase in third births among women aged 35–39 at implementation, suggesting a lasting effect on fertility.

## The Reform in Context

Our sources of exogenous variation are two regional reforms, implemented in 1989–1990, that substantially improved the economic conditions of families and individuals in Northern Troms and Finnmark. The reforms were targeted at recruiting and retaining high skilled labor to the region and to improving the labor market for low skilled workers as part of a long tradition—with considerable political and public opinion support—of “district policies” aimed at maintaining population levels in remote parts of the country. Comparable reforms in other countries were implemented with the explicit aim of increasing fertility (Cohen et al., 2013; Milligan, 2005). When such intentions are explicit, effects on fertility can be mediated by norms and values, above and beyond economic circumstances (Jagannathan et al., 2010). Given that the aim for this reform was to impact internal migration, rather than to affect fertility, changes in norms and values are less likely as mediators.

This section gives a detailed picture of how the increased child benefits (Sect. 2.2) and the tax deductions (Sect. 2.3) changed the economic conditions of women in the treatment region. In Sect. 2.4, we discuss other changes that could have affected fertility differently in the reform and control region, and how we handle these. As a background, we start with a brief description of the fertility level and institutional support to families in Norway at the time.

### Fertility and Institutional Support in Norway Around 1990

During the 1970s, Norway, like most Western countries, saw a sharp decline in total fertility rate (TFR), from 2.5 children per woman in 1970 to 1.72 children per woman in 1980. This co-occurred with an increase in female labor force participation and educational attainment, spurring interest in the potential of institutions to ease the combination of paid work and child rearing. At implementation, important components of what was later to be termed the “Nordic model” of family support was not yet in place in Norway. 18 weeks of parental leave was introduced in 1977. From 1987 to 1993, the length of parental leave was gradually expanded to 42 weeks. In 1993, 4 weeks of paternity quota was introduced (Norwegian Ministry of Children & Families 1996, p. 215). The reform happened prior to the large Norwegian kindergarten expansions: In 1992, 24% of 2 year olds, and 54% of 5 year olds, were in public childcare. In comparison, 42% of 2 year olds and 24% of 5 year olds were looked after by their parents.

At the time, transfers constituted more than half of the public spending on children under school age in Norway (Norwegian Ministry of Children & Families 1996, p. 191). As such, the expansion of the cash transfers for children moved one of the main support schemes for Norwegian parents at the time. In 1988, parents of one child received a yearly transfer of 7 188 NOK, approx. 1040 USD (in 1989 dollars, 6.9 USD/NOK).^2^ The support per child was slightly larger for larger families. For instance, a family with two children would receive 14 868 NOK, roughly 2 150 USD, per year (Norwegian Ministry of Children & Families 1996, p. 675).

### The Child Benefit Reform

Starting January 1st 1989, universal child benefits were increased with 2400 NOK yearly in the reform region, an increase amounting to roughly $347. This pertained to a 33% increase in the universal transfers for first born children, slightly less for later borns. In 1990, additional benefits in the reform region increased to NOK 3600 or $575. Due to inflation, the real value of the benefits declined slightly during the 1990 s despite a small nominal increase in 1991. Neither the general child benefits (for the whole country), nor the additional benefits introduced for Finnmark and North Troms are means-tested.

Anticipatory effects are very unlikely. In August 1988, Prime Minister Gro Harlem Brundtland announced the intention to increase the cash allowance in Finnmark and North Troms, and to generally strengthen the “district policies” targeted at Northern Norway. The final decision was made by the Norwegian Parliament 20th December 1988. The child benefits were paid from January 1st the next year.

**Table 1:** Income increase induced by the regional increase in cash allowance by year and number of children

Year	Childless	1 child	2 children	3 children	4 children	5 children
1989	0	2400	4800	7200	9600	12,000
1990	0	3600	7200	10,800	14,400	18,000
1991–1997	0	3792	7584	11,376	15,168	18,960

Source Regions and Municipalities (2004, p. 134 and 436). Not adjusted for price increase

Table 1 shows the increase in household income induced by the reform, by parity and year. The child cash allowance increases linearly in number of children. Table 2 compares the estimated direct cost of childrearing in the child’s first year of living in Norway in 1989 with the cash allowance for a first child in the reform region (column 4) and in the rest of Norway (column 6). Even absent the regional reform, the Norwegian child allowance reduced the immediate direct cost of a child with 41%. When the reform was fully implemented, women in the reform region received a cash allowance covering 58% of the expenses in the child’s first year of life. The 17 percentage points difference is substantial, and should affect the demand for children if it is indeed sensitive to the direct cost of a child. As the costs of raising a child increases with the child’s age, the proportion covered by the child allowance will decrease over time.^3^ However, as parental earnings increase over time (i.e. with parental age) on average, monetary constraints in the short run may be of particular importance.

**Table 2:** The cash allowance and the monetary cost of a first child

	Cost of child^a^	Size of child benefit^b^					
		Treatment region		Control region		Difference	
	NOK	NOK	%	NOK	%	NOK	%
1989	19,320	10,236	53	7 836	41	2 400	12
1990	21,112	12,348	58	8 848	41	3 600	17

^a^Estimates of cost of living from March 1989 made by the National Institute of Consumer Research (http://www.sifo.no/files/standardbudsjett1989mar.pdf). The sum includes expenses to food, clothes, health, toys, and various equipment in the first year of life. Increases in various household expenses, amounting to approximately 100 NOK per month (depending on household size), are not included. The budget does not account for increases in housing cost driven by an additional child. SIFO budgets for 1990 are not available, the 1989 estimate is adjusted upwards for a 4,1% price increase to give the 1990 estimates (https://www.ssb.no/en/kp)

^b^ Source: NOU 1996 p. 134 and 436

For women in their 20 s and 30 s, the additional cash allowance amounted to about 3–5% of median yearly earnings, with the highest relative value found among women in their early 20 s. For teenagers, who rarely have employment as their main activity, the increase constitutes more than a third of the median yearly income (Supplementary Material, Table S1).

### Tax Deductions

From 1 January 1990, substantial regional income tax deductions for all taxpayers were implemented in the reform region, increasing net wages in Nord-Troms and Finnmark.^4^ Tax deductions were adjusted promptly by the tax authorities, giving obvious and immediate effects on paychecks for salaried employees. Additionally, (private sector) employers in the region were exempt from (mandatory) employer contributions to the national social security system, potentially increasing demand for labor. Through higher wages and lower unemployment, the economic situation in the reform region was to be improved.

The income tax breaks consisted of two parts, an increase in the general (lump-sum) deduction available for all taxpayers and lower (marginal) tax rates for incomes at higher levels. The “base tax”, applying to taxable income at all levels, was set 3.5 percentage points below the rate for the rest of Norway. Additionally, the increase in tax for higher tax brackets, starting at 340 700 NOK, was reduced with 4 percentage points in the reform region, compared to the rest of Norway.^5^ In our sample of women aged 20–39, the threshold is above the 99th percentile of the income distribution, so this reform is of little practical relevance. The difference between the tax levels in the reform and control regions remained relatively stable over time (Angell et al. 2012, p. 140). In sum, the *relative* increase in economic resources was by far highest for individuals with lower earnings, the absolute increase in disposable income increased with (taxable) earnings.

The general deduction was increased by 10 000 NOK per individual, and twice this amount for single parents (Norwegian Ministry of Finance, 1990). The deducted amount of the income is not taxed at any rate, but it cannot become negative tax (i.e. a transfer). The deduction was later increased to 15 000 NOK, again with twice the amount available for single parents (Norwegian Ministry of Finance 2004, p. 9).^6^ The deduction applied to all taxpayers in the region, with and without children. It is applied individually also to married and cohabiting couples.^7^ To be defined as a single parent and receive a double deduction, one must have children below age 18, and neither be married nor in a lasting cohabiting relationship. These differential changes in tax by union status are important for our purpose, as the double deduction substantially reduced the cost of raising a child alone.

### Other Relevant Regional Changes

Starting in 1988, individuals had their (public) student loans reduced by 10% (up to a limit of NOK 15.000) for each year residing and working in Northern Troms and Finnmark, aiming to retain and recruit highly educated individuals. Strictly speaking, the reduced student loan repayments increase disposable income both in the short and long run. While these changes are small and non-transparent, we cannot rule out a priori that they increased educational enrollment and thereby led to fertility postponement (Lappegård & Rønsen, 2005). We test for the presence of such an “enrollment effect” in Sect. 6.

## Theory and Background

### Empirical Studies

In the following, we outline some details on the nature, context and limitations of relevant studies. The expectation from theory is that cash transfers have a positive impact on fertility, while the expected effect of tax reform is ambiguous and depends on the nature of the reform and the preference of those affected. The overall impression from the small number of studies has credibly estimated the causal effect of universal transfers on fertility is a positive relationship (Cohen et al., 2013; González, 2013; Milligan, 2005; Riphahn & Wiynck, 2017).

The Canadian province Quebec has some regional discretion in development of family policies, which has inspired several regional difference-in-difference designs, suggesting positive effects (Ang, 2015; Kim, 2014; Milligan, 2005; Parent & Wang, 2007). It is of some concern that at least some reforms were initiated in part as a response to falling fertility (Ang, 2015; Milligan, 2005), thus making the reform introduction potentially endogenous to the outcome (Besley & Case, 2000). There is also evidence that universal transfers increase fertility in Southern Europe. Using an interrupted time series/RD design, González (2013) finds that a lump sum child benefit of 2 500 EUR increased conceptions in Spain when it was introduced in 2007. She also finds corresponding reductions in maternal labor supply and the propensity to use paid childcare.

Cohen et al. (2013) analyzed the effect of child benefits in Israel, using variation across parities and time for causal identification. They found positive albeit small effects. However, as the benefits were targeted at increasing fertility, reform effects could be mediated through changes in norms and values regarding childbearing, making them less informative of economic fertility effects. The strong pro-natalist sentiments in Israel may also in general compromise the external validity of the estimates to contexts where such sentiments are weaker. As Cohen et al. (2013) mainly utilizes variation in the benefits for the third child, meaningful comparisons of effects across parities are unattainable.

Credible evidence from the Northern European context is limited to Riphahn and Wiynck (2017), who analyze a reform implemented in 1996 in Germany. The reform increased subsidies for first births for high earning couples, and for second births for low earning couples. While results suggest that there is a positive effect on higher earning couples for higher order births, some issues with robustness and counterintuitive results indicate bias from compositional effects.

There is also an extensive literature on fertility effects of target or conditional (cutbacks in) welfare benefits, largely from the US. This includes studies of tax breaks for parents with low income, such as the Earned Income Tax Credit (EITC). Overall, studies with sound causal identification show either no effects, or very small negative effects, on fertility (Dyer & Fairlie, 2004; Fairlie & London, 1997; Joyce et al., 2004; Kearney, 2004; Robins & Fronstin, 1996; Rosenzweig, 1999; Wallace, 2009), see also Bergsvik et al. (2020) for an overview). For tax reforms, the labor supply response is crucial for the fertility effect, with increases in female labor supply tending to go together with negative effects on fertility.

From the Nordic context, Andersen et al. (2018) suggest a postponement effect of increased cash-for-care (CFC) on fertility for some subgroups. CFC is only available for parents of children not in subsidized childcare, and is known to have a lasting negative effect on maternal labor supply (Drange & Rege, 2013). Higher earned income goes together with higher birth rates in the Nordic context (Hart, 2015), and as such extended labor market disruptions is a likely channel for effects. Furthermore, the nature of the CFC reform did not allow for difference-in-difference or regression discontinuity design, making it more likely that results are biased by period trends in fertility.

We also note a related earlier literature using time series data to identify effects of transfers and tax breaks, finding weak or no effects on fertility (for studies on cash transfers see Crump et al. (2011); Ermisch (1988); Gauthier and Hatzius (1997); Kalwij (2010); Walker (1995); Zhang et al. (1994), for an overview of the effects on welfare see Moffitt (1998)). However, studies based on time series data are prone to omitted variable bias from correlated trends in benefits levels and fertility that differ by country or region.

This brief overview of the literature illustrates that a very limited number of studies utilize plausible exogenous variation in economic conditions to estimate effects on fertility behavior. The evidence from Western, secular societies is very limited, and leans heavily on the Canadian province Quebec.

### Theoretical Framework

The reform improved the economic circumstances of (prospective) parents through two channels: increased income (through tax breaks for the employed, and through transfers for parents) and reduced cost of a marginal child (through increased transfers). These changes can influence both fertility and labor supply, and responses to each outcome influence the other. In this section, we outline theoretical perspectives on effects of cash transfers and tax breaks on fertility.

#### Cash Transfers and Fertility

Two fundamental concepts from the microeconomic theory of fertility are crucial to understand the potential effect of the cash transfer increase on fertility: the direct cost of children and the income effect.

In its simplest form, the microeconomic theory of fertility predicts that the demand for children will increase if the cost of raising a child falls (Becker, 1960). The direct cost of a child consists of expenses related to clothes, food, equipment and housing, as well as schooling and health care. Governments can reduce the direct cost of raising a child by cash transfers, tax breaks and housing subsidies, and by providing high-quality public health care and schooling (Gauthier, 2007). The increase in cash transfers in Northern Troms reduces the cost of the next (marginal) child for all women, which is expected to increase fertility.

Furthermore, for mothers, the reform immediately increased the cash transfers for children already born, and thus household income. The simplest micro-economic model of fertility predicts a positive *income effect* on fertility, i.e. that family size increases in household income, all else equal (Becker, 1960). This was later refined to an assumption that the *spending* on children increases in income, but that parents respond to income increases mainly by investing more in each child (Becker & Lewis, 1974). Thus, in our sample, we expect that the increased cash transfer leads mothers to either have more children, or to spend more money on the children they already have (leaving fertility unaffected). In principle, women might also respond to the income increase by reducing their worked hours or even exiting the labor market.

In sum, economic theory rules out that cash transfers have a negative effect on fertility, giving an expectation of either increased or unchanged fertility. The current literature on the effect of cash transfers finds results in accordance with these expectations: reforms increasing cash transfers tend to increase fertility (Cohen et al., 2013; González, 2013; Milligan, 2005), at least at some parities (Riphahn & Wiynck, 2017). In general, the magnitude of the discerned effects is substantial.

#### Tax Breaks and Fertility

The effect of the tax breaks on fertility is more theoretically ambiguous. A tax reduction pertains to an increase in wages, albeit effective immediately only for those already employed. All else equal, an increase in wages increases household income, making for a positive effect on fertility.

However, the all else equal assumption may not hold, as the basic labour/leisure-model suggests that a change in wages is expected to also affect labor supply (hours worked) (Borjas, 2012, pp. 37–39). Effects on labor supply can be positive or negative, depending on individuals’ preferences for leisure (including unpaid work) versus income (and hence paid work). On one hand, some may be willing to work more hours if hourly pay is higher (*substitution effect on labor supply*). On the other hand, the higher wages allow for working fewer hours for the same household income (*income effect on labor supply* (ibid.)). Those who are already employed can respond most quickly to tax changes, so we expect larger effects on overall earnings than on the probability of being employed.

Whether, and if so how, the reform impacted labor supply, can again affect fertility. At the time of the reform, institutional support to families in Norway remained relatively scarce, and mothers of young children often worked part time. In such contexts, women’s labor supply is expected to be negatively related to fertility, as long hours in paid work makes it difficult to find time to care for young children (i.e. a strong *substitution effect on fertility*) (Becker, 1991). Thus, if the tax reform induced women to work more, we expect this to reduce fertility. On the other hand, if the reform led mothers to work less, we expect fertility to increase.

The current literature on tax breaks and fertility is largely based on welfare cutbacks from the US, and find no or small negative effects on fertility (Dyer & Fairlie, 2004; Fairlie & London, 1997; Joyce et al., 2004; Kearney, 2004; Robins & Fronstin, 1996; Rosenzweig, 1999; Wallace, 2009). Of relevance for our study, these negative effects tend to be linked to an increase in female labor supply. However, in the context of US welfare programs, there is also a strong normative aspect in policy implementation, discouraging childbearing while on welfare, which could also contribute to negative effects (Jagannathan et al., 2010). The absence of such normative components in our reform suggests a less negative impact of tax breaks in our study than previously found.

### Expected Effects

In this section, we formulate expectations of how each component of the reform may influence fertility. Theory and the current literature lead to an expectation of a positive effect (or no effect) on fertility from the cash transfer increase. We note that the increased income from cash transfers could lead that hours in paid work and thus gross labor market earnings to fall.

As for tax breaks, pertaining to a wage increase, theory predicts that effects on fertility can be positive or negative. Empirical studies have suggested mainly no or negative effects. The fertility response to the tax break will depend on the labor market response, which cannot be inferred a priori, as it depends on the preference for income versus leisure. Thus, we formulate two contrasting expectations for the effect of tax breaks on labor supply and fertility:

1. Hours in paid work are unmoved $\rightarrow$ net income increases $\rightarrow$ fertility increases
2. Hours in paid work increase $\rightarrow$ fewer hours available for leisure (including childrearing) $\rightarrow$ fertility falls

If we observe a fertility fall, we expect this to be due to the tax break. An increase in fertility, on the other hand, could theoretically be driven by both cash transfers and tax breaks. The empirical literature, however, points to cash transfers as the more likely driver of a fertility increase.

It should be noted that if the reform increased childbearing, it will also be expected to reduce female labor supply (at the ages where fertility is increased) (Angrist & Evans, 1996; Cools & Strøm, 2014). If we observe increased fertility and reduced gross earnings in conjunction, this suggests that the reform increased fertility, and that the fertility response reduced hours in paid work.

#### Effects on Timing Versus Completed Fertility

To assess whether effects are permanent or transitory, we follow the literature in assuming that effects at higher parities and older ages, where the potential for postponement and recuperation is small, indicate quantum effects. As two children was the modal parity in the treated cohorts, effects at parities above two indicate quantum effects.^8^ Furthermore, when effects are assessed over time, a transitory (“timing”) effect would take the shape of an increase in fertility, followed by a fall.

#### Differential Effects by Age

There are some reasons to expect effects to be stronger at young ages. Most importantly, as earnings increase over the life course (Happel et al., 1984), younger women will tend to face stronger monetary constraints than older women. Furthermore, effects at lower ages may regard timing rather than number of children, shown to be more easily influenced by policies (Gauthier, 2007).

#### Differential Effects by Marital Status

The reform constituted a substantially larger improvement of the economic situation for single mothers than for the married (on a relative scale), due to the larger tax breaks. This leads to the naive expectation that effects will be stronger for nonmarital fertility. On the other hand, as living with a partner before having a child has a practical advantage and is to some extent normatively expected (Hobcraft & Kiernan, 1995; Thornton & Young-DeMarco, 2001), one may expect a quicker response to changing economic conditions among women who are living in a formalized union. Furthermore, married women have substantially higher household income than cohabiting and single women (Petersen et al., 2011; Texmon, 1999), and the tax breaks will hence tend to give them the largest improvement in their economic situation on an absolute scale.

## Methods and Data

### Identification Strategy

To identify the effect of the reform, the basic idea is to compare the change in fertility in the treatment region (Northern Troms) to the change in the fertility in the comparison region (Southern Troms). If trends in the two regions are parallel prior to the reform, but differ from the onset of the reform, this indicates a reform effect on fertility.

An alternative way to describe the relationship between economic resources and fertility would be to correlate e.g. earnings, measured at the individual level, with fertility (see e.g. Hart (2015)). A challenge with this approach is that individuals with high earnings differ from individuals with low earnings also in aspects other than earnings that are relevant to fertility—e.g. they may have different preferences and abilities. This gives a risk of omitted variable bias (Angrist & Pischke, 2009). Using an explanatory variable with “quasi-random” variation from policy reforms can handle such omitted variable bias, getting us closer to the causal effect of economic circumstances on fertility (Duncan, 2008). However, as with any methodological approach, ours has limitations. Most importantly, we will capture effects for those who are at the margin of having another child, and for whom money is a relevant constraint. While this does not give a complete picture of the relationship between economic circumstances and fertility, it can be quite informative on the scope for policy change to impact fertility.

### Statistical Model

We estimate difference-in-difference (DD) and event study (ES) models, which pertain to an ordinary least squares (OLS) or linear probability models (LPM) including fixed effects (i.e. a set of dummy variables) for region and year. The fixed effects net out change over time that is shared across regions, and any region specific factors that affects fertility but are constant over time. The main DD-specification takes the following form:

1 $$\begin{aligned} \begin{aligned} Y_{i,t}&=\alpha +\beta _{Ref_{i,t}}X_{Ref_{i,t}}+ \sum _{a=20}^{a=39}\beta_{Age_{i}}X_{Age_{i}}\\&+\sum _{m=1}^M\beta _{Muni_{i,t}}X_{Muni_{i,t}} +\sum _{y=1984}^{1997}\beta _{Year_{i,t}}X_{Year_{i,t}}+\varepsilon _{i,t} \end{aligned} \end{aligned}$$

$X_{Ref}$ is a dummy taking one for reform women in the reform period, so that $\beta _{Ref}$ captures the effect of the reform on the outcome Y. $\beta _{Age}$ are dummy variables for age in years at implementation. $X_{Year}$ are dummies for year, $X_{Muni}$ are dummies for municipality.

If the trends in fertility across the region are similar (absent the reform), Eq. 1 identifies the reform effects. To formally test whether pre-trends are similar, we also estimate event study models (Roth et al., 2022). These models also allow for dynamic effects (i.e. effect variation by year), and take the following basic form:

2 $$\begin{aligned} \begin{aligned} Y_{i,t}=&\alpha +\sum _{dur=-4, dur \ne - 1}^{9}\beta _{Dur_{i,t}}X_{Dur_{i,t}} + \sum _{a=20}^{a=39}\beta _{Age_{i}}X_{Age_{i}}\\&+\sum _{m=1}^M\beta _{Muni_{i,t}}X_{Muni_{i,t}}+\sum _{y=1984}^{1997}\beta _{Year_{i,t}}X_{Year_{i,t}}+\varepsilon _{i,t} \end{aligned} \end{aligned}$$

The variable of interest is now based on a measure of duration since the reform year (1988) in years in the reform group. From this measure, we construct a set of dummy variables taking 1 for each duration, e.g. for observations of women in the reform region in year 1992 the variable $X_{Dur=4}$ takes one, and all other duration dummies take zero. Following convention in event study modelling, women in the comparison group are assigned − 1 on the duration variable, and the dummy for $X_{Dur=-1}$ is the omitted reference category. As the model includes time and municipality fixed effects, as above, the duration variable captures whether, and if so how, the trend in the reform region deviates from the trend in the comparison region, year by year.

The dummy variables for negative values of duration, i.e. the pre-trend, provide a formal, indirect test of the assumption of parallel trends. We perform an F-test of the joint significance of the three estimated dummy variables for the pre-period (duration − 4, − 3, − 2).

Standard errors are robust with clustering on municipality (Angrist & Pischke, 2009). As our number of clusters is relatively small, we use the Wild Cluster Bootstrap to avoid underestimating standard errors (Cameron & Miller, 2015; Roodman, 2015).

To the extent that there is some imbalance between the reform and comparison group, it is useful to re-weigh the sample, so that observations in the comparison group that are comparatively similar to the reform group are given more weight in the regression analysis. Thus, as a robustness test, we also present Inverse Probability Weighted estimates. These serve the same purpose as covariate adjustment (Roth et al., 2022).

### Data and Study Sample

Our data comes from various register sources with information on the full population of Norway, and are merged using a unique person identifier (PIN). Population registers include information on date of birth (for both mothers and children) and municipality of residence as of January 1st each year. Information on educational enrollment and completion was obtained from the National Educational Database (NUDB).

Due to a tax reform implemented in 1983 that affected the treatment and reform region differently, our observation window starts in 1984. Our observation window is limited upwards by the introduction of the cash-for-care reform in 1998, which could change the economic incentives of childbearing and confound the effects of our reform (Andersen et al., 2018). The sample is further restricted to women aged 20–39 in 1988. This ensures that all included women are both old enough to have fertility trends observed prior to the reform, and young enough to have relatively high fecundability at the time of the reform. The sample is further restricted to women born in Norway, and who have at least one parent who is a Norwegian citizen.

**Fig. 1:**
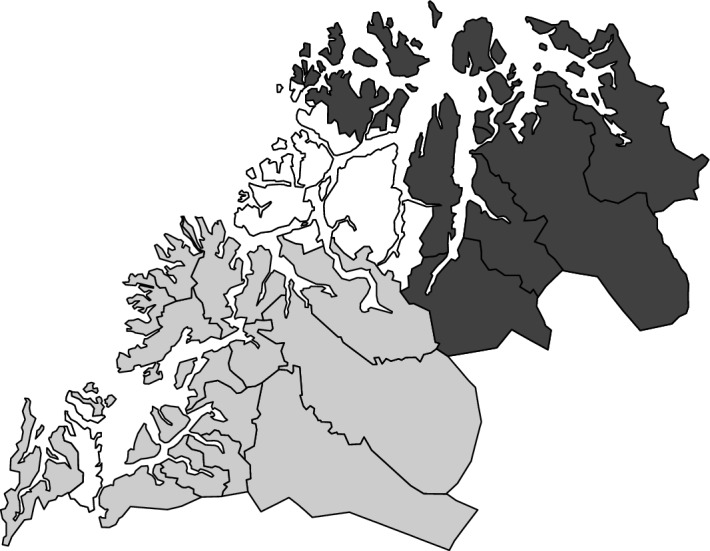
Map of Troms with municipality borders. Treatment municipalities in dark grey, control municipalities in light grey, cities omitted from the control region in white

Our sample is restricted to individuals who lived in either Northern or Southern Troms in 1988. By fixing the sample prior to the reform, we avoid that our results are biased by selective migration. Thus, our estimates are intention to treat (ITT) estimates. The seven reform municipalities in Northern Troms (see Fig. 1) constitute our reform or treatment region.^9^ Our control region is municipalities in Southern Troms. The treatment region has no larger cites, while the cites Tromsø and Harstad are located in Southern Troms. As fertility trends are found to vary on a rural/urban axis (Kulu et al., 2007), we exclude Tromsø and Harstad from our sample. When Tromsø and Harstad are included in the control region, trends are no longer parallel (results available upon request).

### Variables

#### Fertility Outcomes

Our main dependent variable is based on birth dates for all children ever born. For each year, we include children already born, as well as conceptions (imputed as birth date minus average length of pregnancy) leading to live births. From this variable we construct dummy variables for mothers’ parity, i.e. for having at least at least one, two, and three children in the given year.

#### Marital Status

Our data distinguish between married women and unmarried (i.e. cohabiting, single and divorced) women. We construct a dummy variable for being registered as married in the current year. Data from the Medical Birth Registry shows that 80% of the unmarried women were cohabiting with the child’s father at the time of the birth (Supplementary Material, Fig. S.1). However, the share of children who lives with both parents falls with age, and co-residential unions are consistently less stable than marriages (Hart et al., 2017; Lyngstad & Jalovaara, 2010), we consider 20% to be a lower bound for the share of the unmarried who are single parents.

#### Earned Income

Our data includes gross earned income as reported to the tax authorities. The variable includes both labor market earnings and other pensionable transfers.^10^ Pensionable transfers include sick pay, parental leave compensation, and pensions, but excludes social benefits and cash transfers. We refer to this outcome as earnings for brevity. From this variable, we construct two measures:

1. A dummy variable for being employed. The variable takes 1 for women who earn at least the “ base amount” (G) used to adjust pensions and transfers,^11^ otherwise zero. In 1984, the “ base amount” was 22 600 NOK (3 275 USD at with exchange rate 6.9).
2. The natural logartim of earnings. Ajusted to 1984 value using the “base amount”.

#### Educational Attainment and Enrollment

We construct a set of dummies for educational attainment, distinguishing between mandatory (primary and lower secondary), high school and higher education. Missing education is included as a separate category. We also construct a dummy variable for being enrolled in education, taking one if the individual is registered as enrolled in education in the current year.

## Main Results

### Descriptive Results

#### Balancing Tests

As a first step towards establishing whether the reform and comparison region are similar, Table 3 shows means of outcomes and background variables prior to the reform, separately for the reform region (Northern Troms) and the comparison region (Southern Troms). The final column shows the difference between means, and indicates whether the difference is statistically significant. The Table shows that women in Northern Troms are significantly (yet not substantially) less likely to be married, and have lower educational attainment, compared to women in Southern Troms. On average, women in Northern Troms have 0.05 children more than women in Southern Troms prior to the reform. There are no significant differences between the regions in the share in education, the share with personal income, and log income.

**Table 3:** Summary statistics and balance tests

	Northern troms	Southern troms	Difference
Woman’s age	30.28	30.38	− 0.10
Observations	31,980		
Number of children	1.40	1.35	0.05***
Observations	31,980		
Married	0.46	0.48	− 0.02***
Observations	31,980		
In education	0.11	0.11	− 0.01
Observations	31,980		
Has higher education	0.11	0.14	− 0.03***
Observations	31,980		
Log personal income	9.11	9.08	0.03
Observations	31,980		
Has personal income	0.65	0.66	− 0.00
Observations	31,980		

$$^{\dagger }$$$$p<0.10$$, *$$p<0.05$$, **$$p<0.01$$, ***$$p<0.001$$ Sample is women aged 20–39 who lived in the Treatment or Control region in Troms in the period 1984–1997. Characteristics are measured pre-reform (1988)

Our identification strategy handles mean differences between the regions prior to the reform, but requires that the *trends* for the relevant outcomes are parallel. Thus, we proceed to test, first by visual inspection (Sect. 5.1) and then formally in event study models (Sect. 5.2) whether trends in outcomes are parallel in the reform and comparison region prior to the reform.

#### Inspection of Trends

**Fig. 2:**
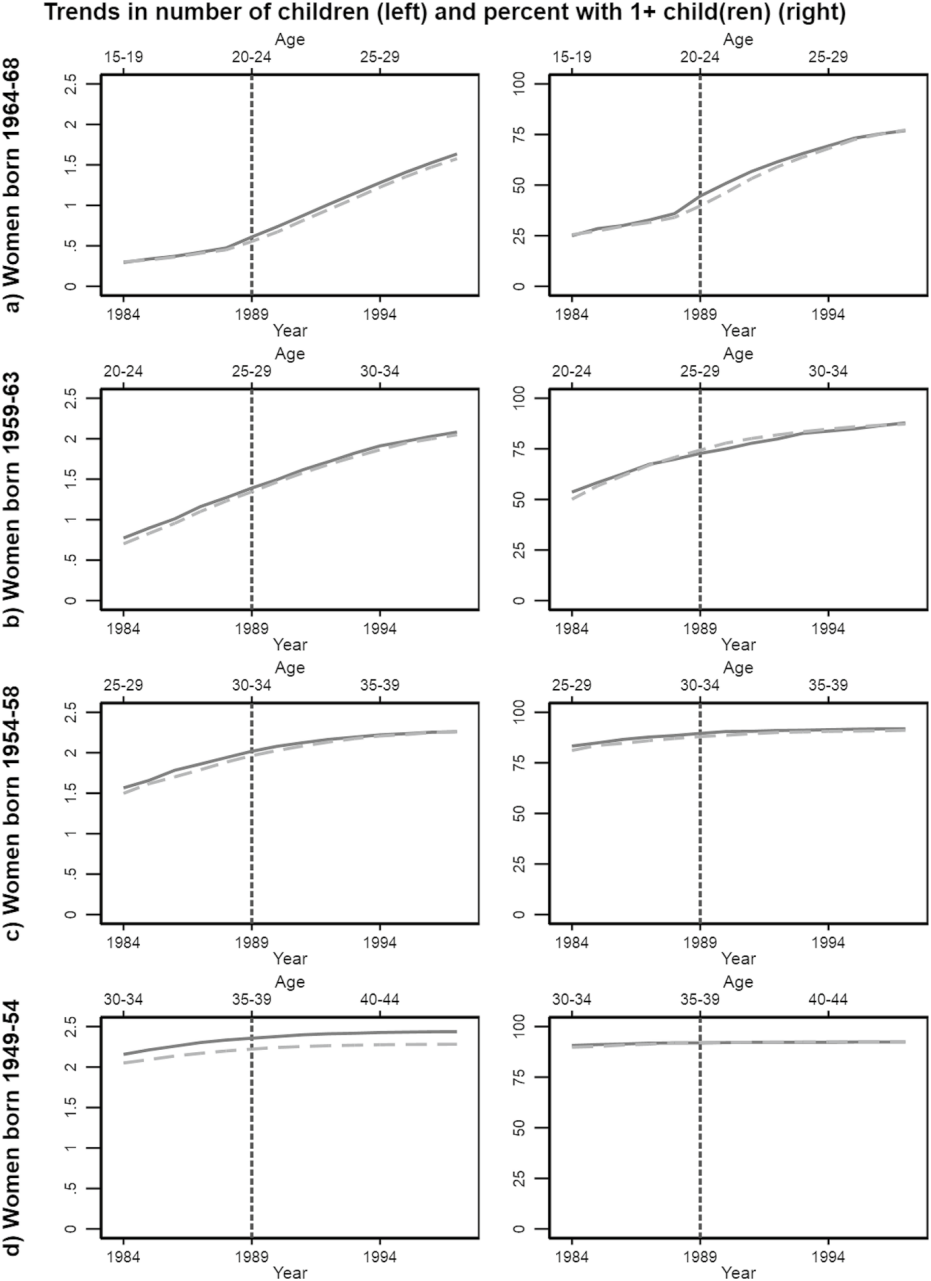
Trends in number of children and the share with at least one child in Northern Troms (full lines) and Southern Troms (dashed lines). Separate plots for four birth cohorts of women

Figure 2 shows the average number of children (left panels) and the share with at least one child (right panels) by calendar year in the reform region (full lines) and the control region (dashed lines). There is a tendency of differential trends by birth cohort (age at reform); therefore, we consistently show results separately by birth cohort.

**Fig. 3:**
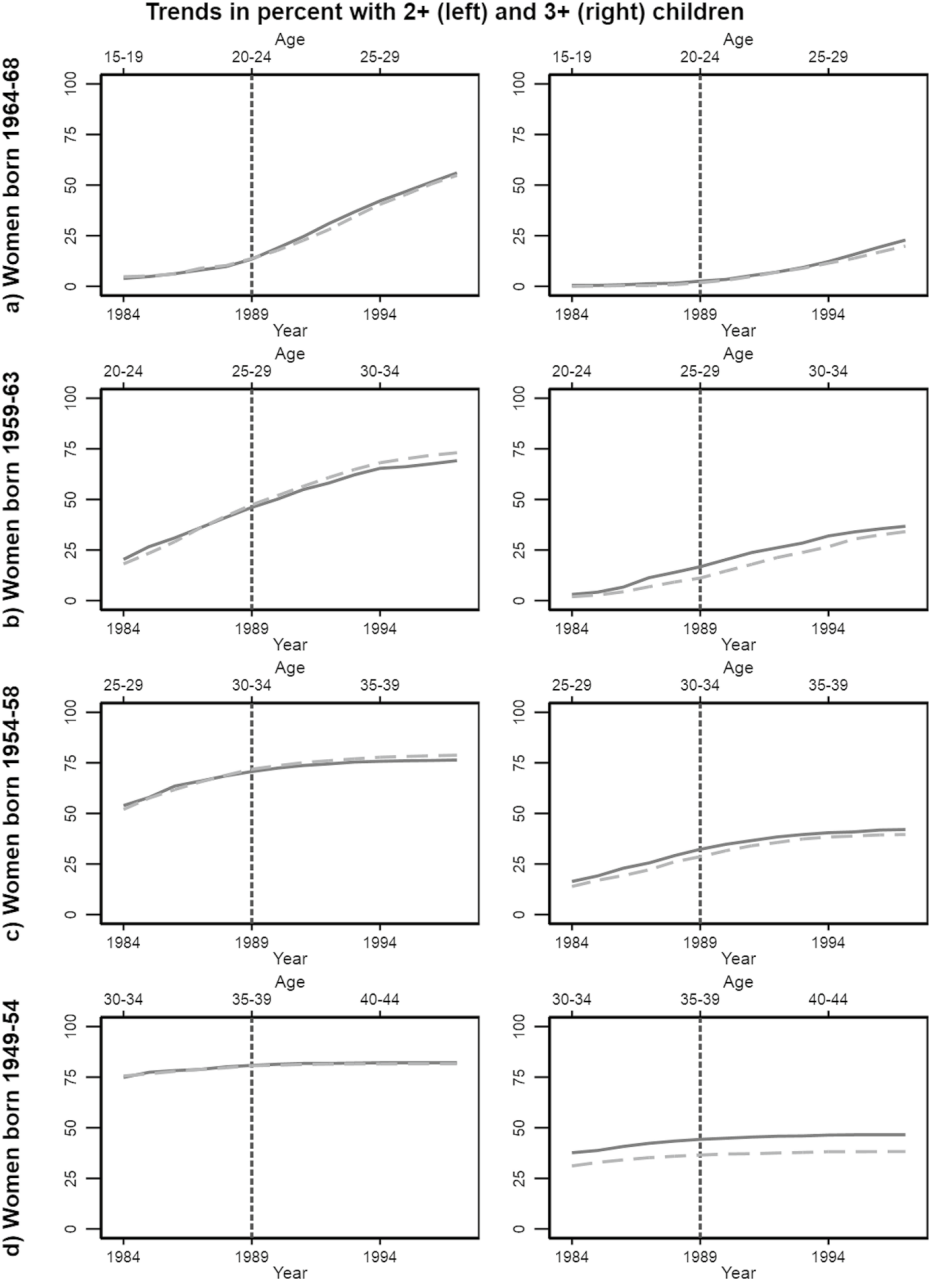
Trends in the share with at least two (left panels) and three (right panels) children in Northern Troms (full lines) and Southern Troms (dashed lines). Separate plots for four birth cohorts of women

Prior to implementation, trends tend to be parallel for both number of children and the share with one child (Fig. 2) and the share with two and three children (Fig. 3) across cohorts, albeit with some notable exceptions: For women aged 25–29 at implementation (panel B) trends are not paralell prior to reform for any outcomes, and for women aged 35–39, trends are not parallel for the share with a least one child. The results for these groups should thus be interpreted with some caution.

The development in trends after implementation can also hint at whether fertility is affected by the reform. After the reform, there is an immediate jump in the share with one child for the youngest cohort (Fig. 2, panel A), followed is followed by a relative increase in the share with two children in the reform region (Fig. 3, panel A). Then, towards the end of the reform period, there is a relative increase in the share of women with three children in the reform region. Thus, the trends suggest a reform effect at all observed parities in this cohort. There is a tendency of relative increases number of children in third births among women aged 35–39 after implementation (Fig. 2, left of panel D), driven by an increase in third births (Fig. 3, right of panel D). As this cohort nears the end of their reproductive agespan, this suggests an impact on completed fertility.

To assess formally whether pre-trends and reform effects are statistically significant, we now move to results from multivariate models.

### Multivariate Results

In this subsection, we present results from multivariate models in two different specifications: Event study models and difference-in-difference estimates. Throughout, we continue to present results separately by women’s birth cohort, due to the observation made above that trends in fertility vary with birth cohort.

#### Event Study Estimates

Event study estimates show, year by year, whether the change in fertility in the reform region deviates significantly from the development in the comparison group.

**Fig. 4:**
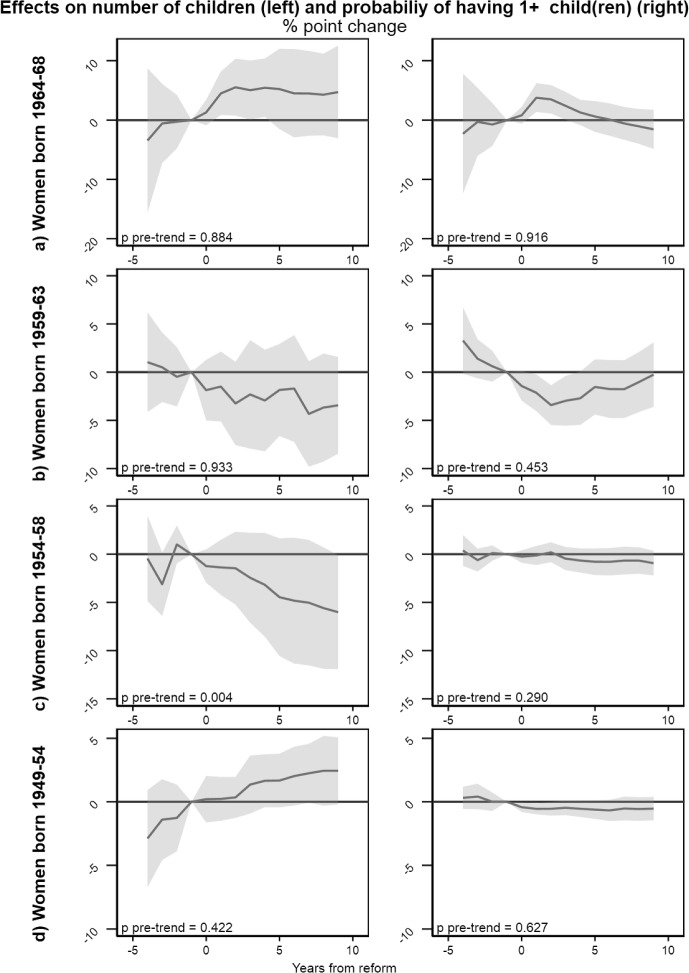
Event study estimates for effects of number of children, and the probability of having at least one child. Separate estimates by birth cohort. Shaded areas indicate 95% confidence intervals. Standard errors are clustered at the municipality level using the wild cluster bootstrap. *P*-values give the result of joint tests of the statistical significance of the estimates in the pre-period. Estimates are controlled for dummy variables for age and calendar time in years, dummy variables for municipality of residence in 1988

**Fig. 5:**
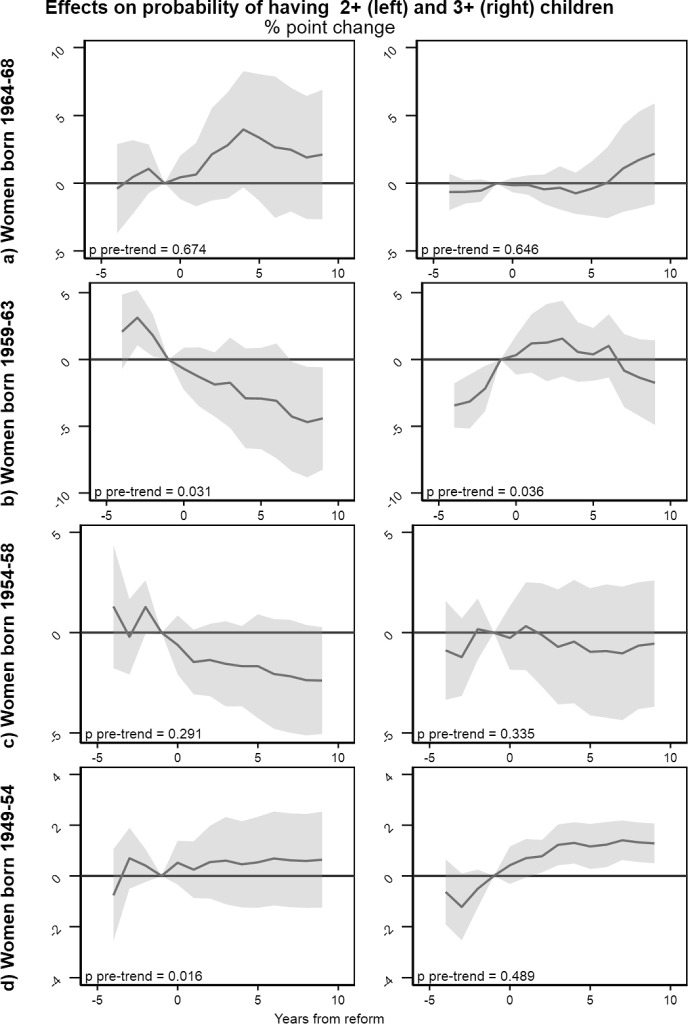
Event study estimates for the probability of having at least two (left panel) and three (right panel) children. Separate estimates by birth cohort. Standard errors are clustered at the municipality level using the wild cluster bootstrap. *P*-values give the result of joint tests of the statistical significance of the estimates in the pre-period. Estimates are controlled for dummy variables for age and calendar time in years, dummy variables for municipality of residence in 1988

In the youngest cohort, there is a relative increase in number of children just after the reform in the reform region, persisting throughout the 10 years we observe (Fig. 4, left panel A). The increase is significant in a short period after implementation, but estimates lose precision and statistical significance over time. There is also a significant increase in the share of women with one child in the years immediately after the reform (right panel A). Effects for higher parities, we see the tendency of a lagged increase in first the share with two children, and then the share with three children, in the reform region (Fig. 5, Panel A). While precision is limited and estimates are not statistically significant, this indicates that the increase in number of children in the youngest cohort is due to driven by changes at all parities. For all outcomes this group, the estimated coefficients for pre-trends are close to zero, and the joint test of the statistical significance of pre-trend is far from significant, supporting that the assumption of parallel trends holds.

For women aged 25–29 at implementation, event study estimates show no effect on number of children, and a slight decrease in the share with at least one child (Fig. 4, panel B). However, the pre-trend estimates suggest that this decrease is part of a trend that started prior to implementation, so that it should not be interpreted as a reform effect. For higher parity transitions pre-trends are noisy and often statistically significant, indicating that the parallel trend assumption does not hold in this subsample (Fig. 5, Panel B).

Turning to women aged 30–34 years at implementation (Figs. 4 and 5, panel C), pre-trends are consistently noisy, and statistically different from zero when the outcome is number of children. While we find no statistically significant reform effects in this age group, the estimated pre-trends suggest that the assumptions for causal inference do not hold in this age group.

Finally, for women aged 35–39 at implementation (panel D, right), there is a tendency of increase in number of children (Fig. 4, left panel D) and a statistically significant relative increase in the share with three children (Fig. 5, right panel D). Statistically insignificant pre-trends indicate that the increase could have started prior to the reform. However, we interpret this as indicative evidence of an increase in completed family size in the oldest age group due to the reform.

#### Difference-in-Difference Estimates

To summarize the magnitude of the estimated effects, we show difference-in-difference estimates in Table 4. For completeness, we show results for all cohorts and outcomes displayed in the event study models in Sect. 5.2. However, our substantive interest lies in the cohorts where pre-trend inspection suggests that the identifying assumption holds, that is, among the youngest and oldest women. Given indications of dynamic effects, we show results both for the full observation period (up to 1997, upper panel), and for a shorter period (up to 1993, lower panel).

**Table 4:** Difference-in-difference estimates of effects on fertility outcomes

(a) Full panel (1984–1997)				
	(1)	(2)	(3)	(4)
	Ages 20–24	Ages 25–29	Ages 30–34	Ages 35–39
Number of children	0.048	$-$ 0.026	$-$ 0.031	0.027
	[$-$ 0.011, 0.12]	[$-$ 0.085, 0.034]	[$-$ 0.098, 0.049]	[$-$ 0.012, 0.067]
Observations	34,310	31,458	31,682	29,470
1 or more child(ren)	0.010	$-$ 0.027	$-$ 0.005	$-$ 0.006
	[$-$ 0.017, 0.036]	[$-$ 0.072, 0.0095]	[$-$ 0.018, 0.012]	[$-$ 0.017, 0.0060]
Observations	34,310	31,458	31,682	29,470
2 or more children	0.021	$-$ 0.043	$-$ 0.022	0.004
	[$-$ 0.016, 0.059]	[$-$ 0.085, $-$ 0.00024]	[$-$ 0.048, 0.0035]	[$-$ 0.014, 0.025]
Observations	34,310	31,458	31,682	29,470
3 or more children	0.006	0.019	$-$ 0.001	0.015
	[$-$ 0.014, 0.030]	[$-$ 0.0050, 0.050]	[$-$ 0.035, 0.034]	[0.0030, 0.025]
Observations	34,310	31,458	31,682	29,470

(b) Short panel (1984–1993)				
	(1)	(2)	(3)	(4)
	Ages 20–24	Ages 25–29	Ages 30–34	Ages 35–39
Number of children	0.050*	$-$ 0.022	$-$ 0.018	0.021
	[0.0011, 0.11]	[$-$ 0.082, 0.038]	[$-$ 0.079, 0.049]	[$-$ 0.015, 0.057]
Observations	22,794	22,470	22,630	21,050
1 or more child(ren)	0.024	$-$ 0.033*	$-$ 0.003	$-$ 0.006
	[$-$ 0.0025, 0.048]	[$-$ 0.073, $-$ 0.00043]	[$-$ 0.017, 0.012]	[$-$ 0.016, 0.0048]
Observations	22,794	22,470	22,630	21,050
2 or more children	0.022	$-$ 0.034	$-$ 0.019	0.003
	[$-$ 0.013, 0.062]	[$-$ 0.075, 0.0055]	[$-$0.042, 0.0044]	[$-$ 0.012, 0.021]
Observations	22,794	22,470	22,630	21,050
3 or more children	$-$ 0.002	0.027	0.001	0.014*
	[$-$ 0.012, 0.011]	[$-$ 0.0010, 0.057]	[$-$0.032, 0.034]	[0.0015, 0.025]
Observations	22,794	22,470	22,630	21,050

Estimates are controlled for dummy variables for age and calendar time in years, dummy variables for municipality of residence in 1988. Standard errors are clustered at the municipality level using the wild cluster bootstrap

In the full panel, for the youngest cohort, the point estimate suggest that the reform increased number of children with 4.8 percentage points, but is not statistically different from zero. Point estimates for the other cohorts, and for other parities, are also statistically insignificant at the 5 percent level.

In the shorter panel, point estimates suggest a 5 percentage point increase in number of children in the youngest cohort (p<0.05). Point estimates for the share with one and two children are also positive, albeit not statistically significant, in this cohort. There is also, in line with the event study plots, a 1.4 percentage points increase in the share with three children in the oldest cohort (*p*<0.05). With one exception, effects for all other outcomes and samples are statistically insignificant.^12^

## Mechanisms and Robustness Tests

### Effects by Marital Status and on the Propensity to Marry

As outlined above, single mothers will benefit more from the reform on a relative scale, while married and cohabiting women may be in a union context where they can more readily respond to changed economic incentives. Our data allows us to distinguish between married and unmarried mothers, with the latter group consisting of both cohabiting and single mothers.

**Table 5:** Difference-in-difference estimates of effects on fertility outcomes. Separate estimates by marital status at implementation. Short panel (1984–1993)

(a) Women unmarried at implementation				
	(1)	(2)	(3)	(4)
	Ages 20–24	Ages 25–29	Ages 30–34	Ages 35–39
Number of children	0.047	$-$ 0.032	$-$ 0.040	0.003
	[$-$ 0.00030, 0.10]	[$-$ 0.10, 0.044]	[$-$ 0.11, 0.035]	[$-$ 0.11, 0.075]
Observations	20,823	13,650	8010	4800
1 or more child(ren)	0.023*	$-$ 0.053**	$-$ 0.002	$-$ 0.008
	[0.0025, 0.047]	[$-$ 0.095, $-$ 0.020]	[$-$ 0.036, 0.047]	[$-$ 0.046, 0.022]
Observations	20,823	13,650	8010	4800
2 or more children	0.023	$-$ 0.006	$-$ 0.020	$-$ 0.002
	[$-$ 0.011, 0.059]	[$-$ 0.052, 0.045]	[$-$ 0.056, 0.051]	[$-$ 0.041, 0.042]
Observations	20,823	13,650	8010	4800
3 or more children	$-$ 0.003	0.021	$-$ 0.001	0.028
	[$-$ 0.012, 0.0066]	[$-$ 0.0070, 0.062]	[$-$ 0.051, 0.034]	[$-$ 0.020, 0.063]
Observations	20,823	13,650	8010	4800

(b) Women married at implementation				
	(1)	(2)	(3)	(4)
	Ages 20–24	Ages 25–29	Ages 30–34	Ages 35–39
Number of children	0.054	0.017	$-$ 0.004	0.023
	[$-$ 0.21, 0.23]	[$-$ 0.094, 0.15]	[$-$ 0.088, 0.088]	[$-$ 0.021, 0.080]
Observations	1971	8820	14,620	16,250
1 or more child(ren)	0.004	$-$0.013	$-$ 0.004	$-$ 0.006
	[$-$ 0.12, 0.11]	[$-$ 0.066, 0.036]	[$-$ 0.021, 0.012]	[$-$ 0.012, 0.00016]
Observations	1971	8820	14,620	16,250
2 or more children	$-$ 0.008	$-$ 0.067*	$-$ 0.018	0.002
	[$-$ 0.11, 0.082]	[$-$ 0.12, $-$ 0.014]	[$-$ 0.052, 0.017]	[$-$ 0.012, 0.017]
Observations	1971	8820	14,620	16,250
3 or more children	0.022	0.052	0.003	0.010
	[$-$ 0.099, 0.12]	[$-$ 0.014, 0.13]	[$-$ 0.039, 0.049]	[$-$ 0.0077, 0.028]
Observations	1971	8820	14,620	16,250

Estimates are controlled for dummy variables for age and calendar time in years, dummy variables for municipality of residence in 1988. Standard errors are clustered at the municipality level using the wild cluster bootstrap

We estimated difference-in-difference estimates separately by marital status prior to the reform, shown in Table 5. For the youngest age group, there is a tendency of effects being driven by the unmarried sample, with a statistically significant 2.3 percentage points increase in the share with at least one child among unmarried women. Separate event study plots by marital status support this pattern. For married women aged 20–24 at implementation, there are no significant effects (Supplementary Material, Fig. S.3). For women unmarried at implementation, there is a significant increase in both number of children, and the share of women with at least one child, immediately following the reform (Fig. S.4). For higher order births (available upon request), event study estimates by marital status are very imprecise and reveal no clear pattern.

It is possible that the unmarried women who were moved by the reform to have a first child, also were moved to marry. We tested this empirically by estimating reform effects on the propensity to marry (Table 6), but found no significant effects in any sample. This indicates that the reform increased non-marital fertility in the youngest cohort.

**Table 6:** Difference-in-difference estiamtes for alternate outcomes. Separate estimates by birth cohort

Married	$-$ 0.010	0.012	0.006	0.015
	[$-$ 0.037, 0.023]	[$-$ 0.037, 0.057]	[$-$ 0.028, 0.042]	[$-$ 0.021, 0.049]
Observations	34,310	31,458	31,682	29,470
In education	0.043	0.006	0.009	$-$0.009
	[$-$ 0.0086, 0.092]	[$-$ 0.026, 0.043]	[$-$ 0.0025, 0.022]	[$-$0.024, 0.0089]
Observations	34,310	31,458	31,682	29,470
Has higher education	$-$ 0.044	$-$ 0.010	0.007	0.002
	[$-$ 0.064, $-$ 0.026]	[$-$ 0.036, 0.013]	[$-$ 0.0053, 0.019]	[$-$ 0.012, 0.016]
Observations	34,310	31,458	31,682	29,470
Log personal income	$-$ 0.008	0.089	0.029	$-$0.294
	[$-$ 0.42, 0.35]	[$-$ 0.29, 0.39]	[$-$ 0.31, 0.35]	[$-$ 0.69, $-$ 0.017]
Observations	34,310	31,458	31,682	29,470
Has personal income	$-$ 0.021	0.005	0.012	$-$ 0.016
	[$-$ 0.086, 0.031]	[$-$ 0.050, 0.058]	[$-$ 0.031, 0.052]	[$-$0.061, 0.020]
Observations	34,310	31,458	31,682	29,470

### Earned Income and Educational Enrollment

The reform also incentivized women to enroll in education and work more paid hours, potentially reducing fertility and counteracting a positive effect of universal transfer on fertility. To test whether these mechanisms were at work, we estimated reform effects on log earned income, the probability of having earned income, and educational enrollment and attainment (Table 6). As above, we estimate effects separately by birth cohort. We do not find significant effects on any of these outcomes in our sample. Event study plots for these outcomes (Supplementary Material, Figs. S.7 and S.6) largely corroborate this pattern.^13^

In short, changes in earnings, employment, and educational enrollment and attainment, all known to be important fertility determinants, are unlikely to explain the effects on fertility estimated above.

### Inverse Probability Weighing

Balance tests (Table 3) indicated some imbalances between the reform and comparison groups on observable characteristics. Inverse probability re-weighing account for such imbalances, and yield qualitatively similar results (Supplementary Material, Fig. S.8 and Table S.2).

## Concluding Discussion

While previous studies have demonstrated convincingly that the low indirect costs of childbearing matters for the high fertility in the Nordic countries (Rindfuss et al., 2010), knowledge of the importance of the economic circumstances in the Nordic context has been more scarce. Using credibly causal evidence from Norway, we show that a reform increasing universal transfers and tax breaks increased fertility in the Nordic context. This indicates that the low direct cost of raising a child has contributed to the high fertility in the Nordic region.

Our findings are in line with economic theory, and corroborate a scarce literature of credibly causal studies suggesting a positive effect of transfers on fertility (see Milligan (2005) and Ang (2015) for Quebec, González (2013) for Spain, Cohen et al. (2013) for Israel, and Riphahn and Wiynck (2017) for Germany). The results hinge on identifying assumptions, supported by extensive indirect tests, discussed further below.

Unlike most previous studies, our reform gave significant variation in benefits also for first births. Indeed, our results reveal the largest effect at this parity. More specifically, universal transfers increased first birth rates among young, unmarried women. The stronger effects on younger women are in line with the larger economic constraints at this age (Happel et al., 1984), and also with timing of fertility being more easily affected than quantum (Bergsvik et al., 2021; Gauthier, 2007).

Our results also support quantum effects. There are indications of effects also at higher parities among women in their early 20 s at implementation, and of effects on third births among women aged 35–39 at implementation. Both these findings point to that the reform impacted completed fertility. This is in line with previous studies from other contexts on the effect of transfers on higher-parity fertility (Cohen et al., 2013; Milligan, 2005).

The reform permanently increased non-marital fertility, as the propensity to marry in the same age group was unmoved. The naive expectation that married women would be more easily affected, as childbearing is normatively expected to take place within a union, was thus not supported (Thornton & Young-DeMarco, 2001). Other studies have found that married women respond more efficiently to changes in economic constraints (Cohen et al., 2013), but our reform is distinct in that it reduced the relative cost of non-union childrearing. This added economic security for single mothers seems to have translated into higher non-marital fertility. It may also be relevant that Northern Norway has a tradition for nonmarital childbearing (Noack, 2010), meaning that women outside formalized unions may respond quicker to changes in economic incentives here than in other Norwegian regions.

Our expectation, based on theory and previous research, was responses with respect to earnings and fertility would be intertwined. Detailed full population data allowed us to investigate effects on gross earnings, which we found to be largely unmoved. Thus, the increased (net) wages due to the tax break did not induce women to work longer hours. One explanation for this could be that women in Troms, when facing a wage increase, preferred to work the same number of hours for slightly higher pay, cf. the labor/leisure-model (Borjas, 2012). It is also possible that rigidities in the labor market at the time made responses difficult, e.g. that overtime was not readily available for the full-time employed. We also note that there is some imprecision to the estimates, so that smaller yet economically meaningful changes may not be captured. The absence of negative effects on earnings means that the slight increase in fertility did not lead women to reduce their hours in paid work. In other words, the reform did not weaken young mothers’ commitment to the “dual strategy” of work and motherhood (Ellingsæter & Rønsen, 1996).

The combination of a clean quasi-experimental design and access to population data of high quality gives our study credibility when compared to previous similar setups. We compared women who lived in the same county at the same time, but were exposed to different economic policies. The treatment and control regions consist of municipalities comparable on observable characteristics, and display similar trends in fertility prior to the reform. As our reform is not targeted at increasing fertility, effects mediated through mechanisms other than changes in costs or income are very unlikely. The combination of a regional reform and extremely detailed data allows us to construct an *a priori* plausible control group, and for extensive (indirect) testing of the identifying assumption. The plausibility of our identifying assumption is also strengthened by the estimates being relatively unchanged by inverse probability weighing. The subsample estimations, crucial for the interpretation of our results, are obtained without endogenous conditioning (in contrast to e.g. Cohen et al. (2013) and Milligan (2005)). The indication of selective migration underlines the need of using an exogenous measure for treatment. Particularly, regional reforms targeted at changing fertility (see e.g. Milligan (2005)) may very well induce in-migration of individuals with above-average latent fertility.

Some concerns with our analysis should be mentioned. First, our results hinge on identifying assumptions, and have a causal interpretation as long as these assumptions hold. Our indirect tests of these assumptions suggest that they are more plausible in some groups than others. In particular, in the youngest age groups, the estimated pre-trends are statistically similar, corroborating the validity of our results. Second, while theoretical expectations of how tax benefits and cash allowances interact can be outlined, studying each of these in isolation would make for a theoretically cleaner interpretation of the results. As it is, we are able to test that the reform did not affect women’s labor supply through other channels than increasing fertility (i.e., that the substitution effect does not bias our estimates downwards). However, due to the combination of effects, we abstain from calculating price- and income elasticities, which again complicates precise comparison of reform effects across contexts.

As our main effects are identified among unmarried women, it would be of both theoretical interest and policy relevance to investigate whether the effects are concentrated among non-union births, or births to cohabiting women. Unfortunately, limitations of register data on cohabitation in the sampling period do not permit such investigations. To the extent that single women are affected more strongly than cohabitors, this means that our effects will be diluted and thus biased downwards. We are not aware of survey data set on births to cohabitors that would allow us to both zoom in sufficiently to get a plausible control group, and to retain a sample size sufficient to identify effects of a meaningful size.

As for all quasi-experimental studies, the external validity of estimates remains a concern. With publicly covered high-quality schooling (through university) and nearly free public high-quality health care, the direct cost of a child in Troms in around 1990 was relatively low compared to most other regions in today’s Western world. On the other hand, interest rates were revolving around 10 at the time of the reform,^14^ making financial strain widespread for home owners. Taken together, these economic conditions are in no way exceptional. Our external validity is strengthened by the finding of similar effects in Canada (Milligan, 2005), Spain (González, 2013), Germany (Riphahn & Wiynck, 2017) and Israel (Cohen et al., 2013), suggesting that a positive effect of improved economic conditions on fertility is a general pattern in modern Western societies.

Our results illustrate how changes in the cost of a child can not only influence fertility, but also change the order of life courses, shifting some births from higher ages and formalized unions to lower ages, where unions are less likely to be formalized, or even formed. At the societal level lower mean age at birth can in and of itself have a lasting impact on population structure as generational lengths shorten (Goldstein et al., 2003). An earlier transition to motherhood may facilitate having a larger family, for which we also find some indicative evidence, and prevent health problems at higher-order births. Our results do not indicate that the additional births hampered labor supply, meaning that poverty was an unlikely consequence of the reform. A shift of births from more stable to less stable union contexts may, however, have less favorable consequences to the extent that stability of parental unions has benefits for adults and children.

Our study corroborates that economic conditions affect fertility choice in the Nordic contexts. In context of the current Nordic fertility decline (Hellstrand et al., 2021), our findings emphasize economic security as an important component of the Nordic fertility regime. Recent changes in economic security—linked to declining real values of transfers or increasing housing costs—deserve scrutiny as explanations of the fertility decline.

## Supplementary Information

Below is the link to the electronic supplementary material.

Supplementary file 1 (pdf 1014 KB)

## Data Availability

The article is based on analysis of administrative data. For privacy/data security reasons, these can not be made publicly available.

## Footnotes

1 Using an exchange rate of 6.25 NOK per USD. All conversion rates are obtained from http://www.norges-bank.no/en/Statistics/exchange_rates/currency/USD/.

2 http://www.norges-bank.no/en/Statistics/exchange_rates/currency/USD/.

3 http://www.sifo.no/files/standardbudsjett1989mar.pdf.

4 Prior to 1990, individual taxpayers in the region had enjoyed a slightly higher general tax deduction than taxpayers in the rest of the country. (http://www.regjeringen.no/en/dep/krd/Subjects/rural-and-regional-policy/virkeomrader-retningslinjer-og-regler/action-zone-in-finnmark-and-nord-troms.html?id=527171).

5 This applies to Innslagspunkt I, i.e. the lowest of the top tax brackets (Norwegian Ministry of Regions & Municipalities 2004, p. 9).

6 The deduction was 10 000/15 000 NOK in Tax Class I, and 20 000/30 000 NOK in Tax Class II. Single parents (“enslige forsørgere”) are classified as Tax Class II.

7 https://www.skatteetaten.no/en/person/taxes/get-the-taxes-right/family-and-health/distributing-income-expenses-and-capital/.

8 Statistics Norway StatBank https://www.ssb.no/en/statistikkbanken, Table 05769.

9 These are Kvernangen, Skjervøy, Nordreisa, Kåfjord, Lyngen, Storfjord and Balsfjord.

10 https://www.ssb.no/a/metadata/conceptvariable/vardok/7/nb.

11 https://www.nav.no/no/NAV+og+samfunn/Kontakt+NAV/Utbetalinger/Grunnbelopet+i+folketrygden.

12 Estimates show a significant decrease in the share of women with at least two children among women aged 25–29 years at implementation. As the event study estimates suggested that pre-trends were not parallel for this outcome and sample, we are reluctant to interpret this as a reform effect.

13 There is a tendency of increased educational enrollment but also lower educational attainment in the younger cohort, but for these outcomes, trends were also far from parallel prior to the reform.

14 https://www.norges-bank.no/pengepolitikk/Styringsrenten/.
