# Supplementary material for: Universal Transfers, Tax Breaks and Fertility: Evidence from a Regional Reform in Norway
Source: Popul Res Policy Rev. 2023 May 23;42(3):49. doi: 10.1007/s11113-023-09793-z (PMC10205036; doi:10.1007/s11113-023-09793-z)
Supplement: Supplementary file 1 — (pdf 1014 KB) [file 11113_2023_9793_MOESM1_ESM.pdf]

Supplementary Material

Table S.1: Transfer as proportion of median income in treatment region by age group in 1988

Age group	Prop median
15-19	.37
20-24	.05
25-29	.03
30-34	.03
35-39	.02

Table S.2: Difference-in-difference estimates of effects on fertility outcomes. Main models estimated with Inverse Probability Weighing.

	(1) Ages 20-24	(2) Ages 25-29	(3) Ages 30-34	(4) Ages 35-39
Number of children	0.048	-0.009	-0.018	0.030
	[-0.016, 0.13]	[-0.065, 0.044]	[-0.092, 0.055]	[-0.0090, 0.068]
Observations	34215	31374	31570	29442
1 or more child(ren)	0.023	-0.010	-0.003	-0.006
	[-0.0063, 0.053]	[-0.055, 0.027]	[-0.017, 0.015]	[-0.017, 0.0048]
Observations	34215	31374	31570	29442
2 or more children	0.016	-0.031	-0.014	0.006
	[-0.019, 0.054]	[-0.068, 0.0099]	[-0.040, 0.015]	[-0.011, 0.026]
Observations	34215	31374	31570	29442
3 or more children	-0.000	0.015	0.004	0.017
	[-0.020, 0.022]	[-0.011, 0.045]	[-0.028, 0.038]	[0.0056, 0.027]
Observations	34215	31374	31570	29442

Note: Estimates are controlled for dummy variables for age and calendar time in years, dummy variables for municipality of residence in 1988.Standard errors are clustered at the municipality level using the wild cluster bootstrap.Inverse Probability Weighing includes covariates for age, marital status, educational attainment, educational enrollment, and age of youngest child, all measured in 1988.

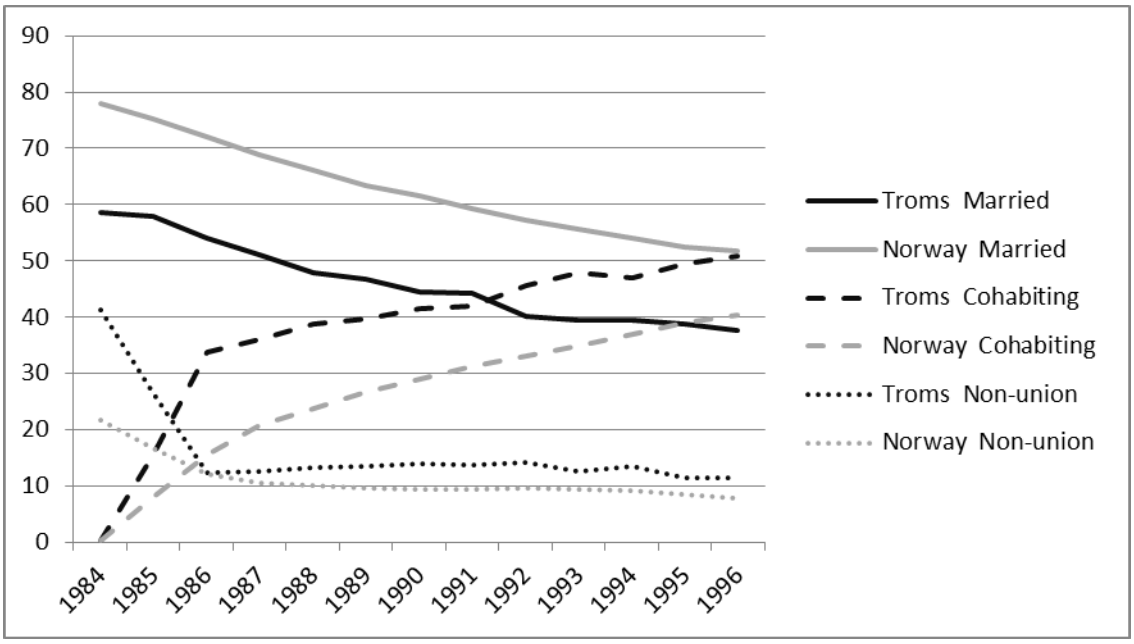

Figure S.1: All births by union type. Troms and Norway.

Source: Data from Medical Birth Register, accessed online at `http://statistikkbank.fhi.no/mfr/`. *F4b, Live births by mothers' union status.* Own calculations. The rapid increase in the proportion births to cohabiting women 1984-87 is mainly due to a correction of underreporting of births cohabiters in this period

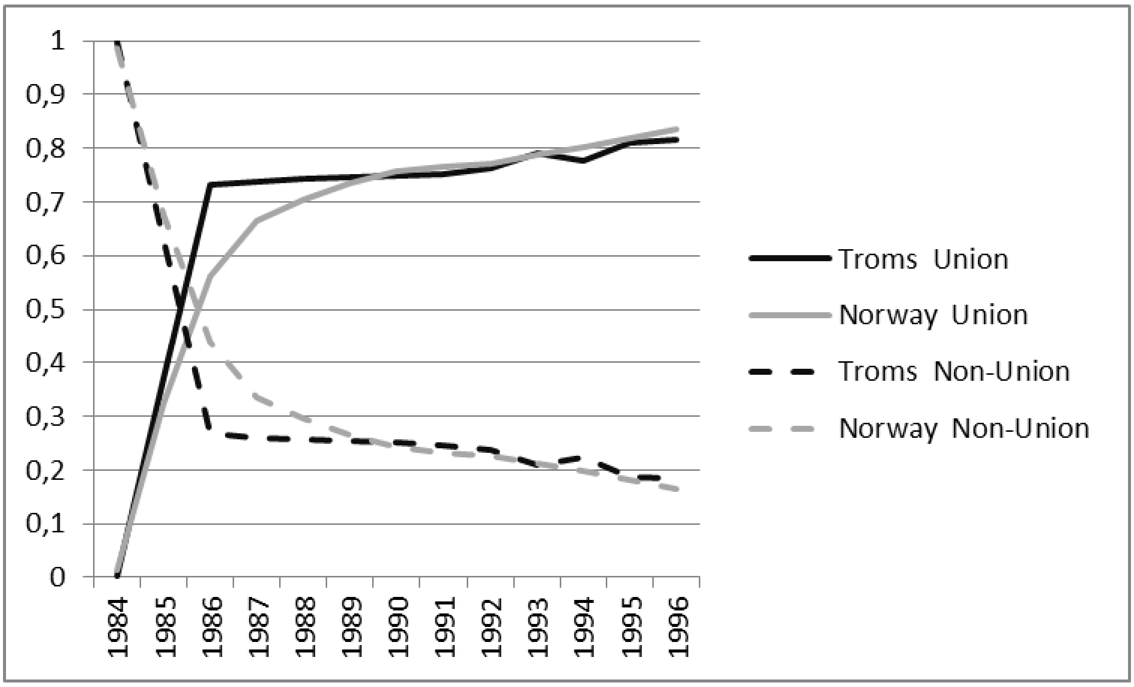

Figure S.2: Non-marital births by union type. Troms and Norway.

Source: Data from Medical Birth Register, accessed online at `http://statistikkbank.fhi.no/mfr/`. *F4b, Live births by mothers' union status.* The rapid increase in the proportion births to cohabiting women 1984-87 is mainly due to a correction of underreporting of births cohabiters in this period

Figure S.3: Event study estimates for effects of number of children, and the probability of having at least one child. Women married at implementation. Separate estimates by birth cohort.

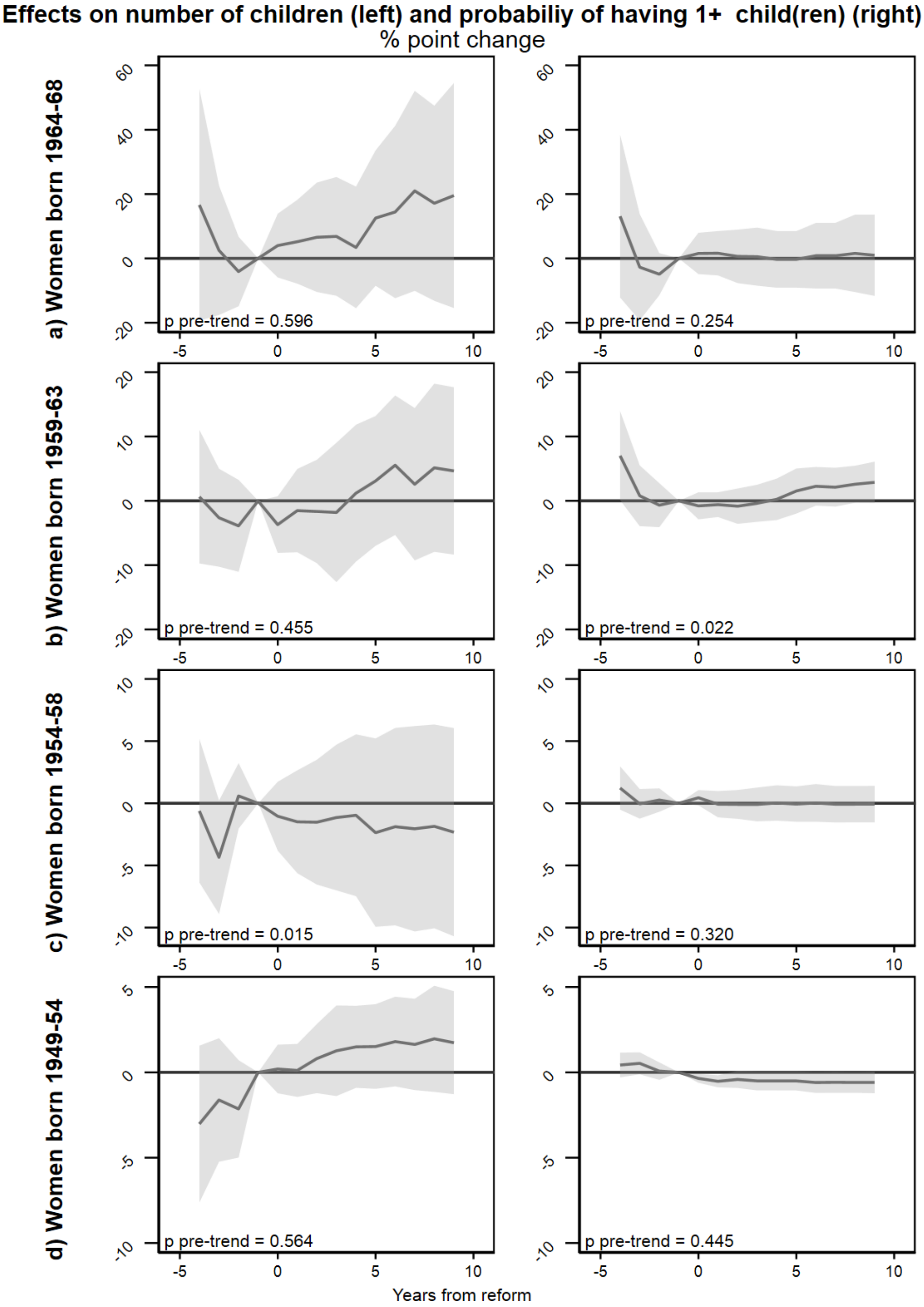

Note: Standard errors are clustered at the municipality level using the wild cluster bootstrap. P-values give the result of joint tests of the statistical significance of the estimates in the pre-period. Estimates are controlled for dummy variables for age and calendar time in years, dummy variables for municipality of residence in 1988.

Figure S.4: Event study estimates for effects of number of children, and the probability of having at least one child. Women unmarried at implementation. Separate estimates by birth cohort.

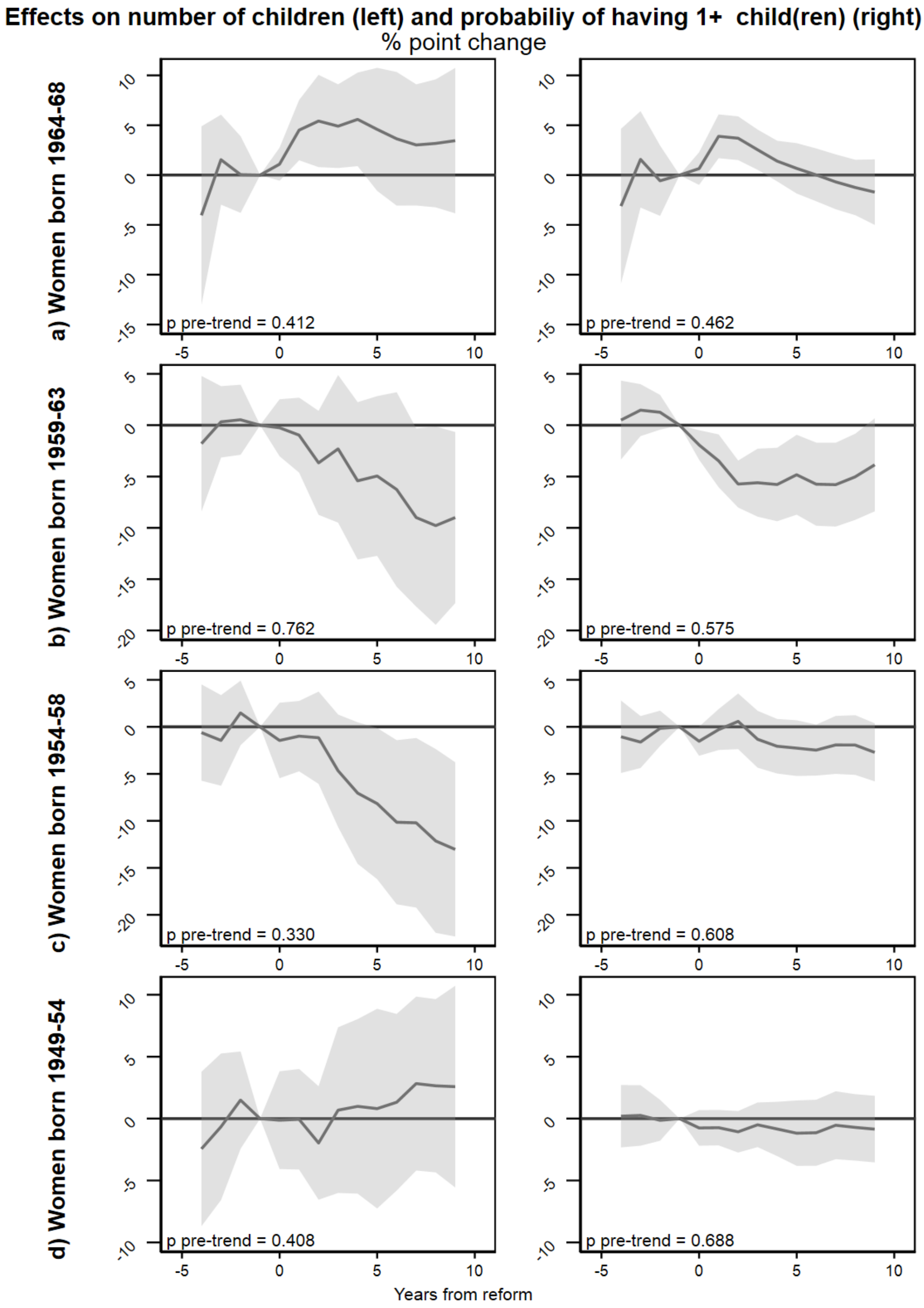

Note: Standard errors are clustered at the municipality level using the wild cluster bootstrap. P-values give the result of joint tests of the statistical significance of the estimates in the pre-period. Estimates are controlled for dummy variables for age and calendar time in years, dummy variables for municipality of residence in 1988.

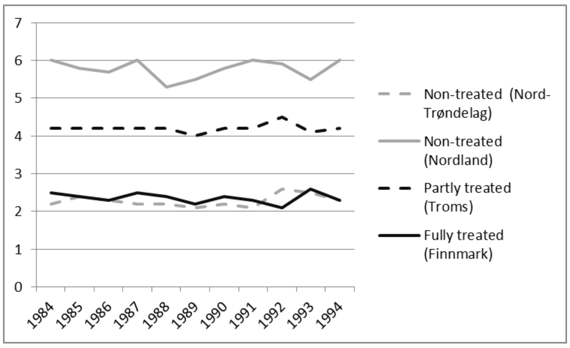

Figure S.5: Induced abortion as percentage of live births. By region and year
Source: The Medical Birth and Abortion Register, accessed online at `http://statistikkbank.fhi.no/mfr/`. *Induced abortions as percentage of all births by region ("fylke").*

Figure S.6: Event study estimates for the probability of being employed (left panel) and log earnings (right panel). Separate estimates by birth cohort.

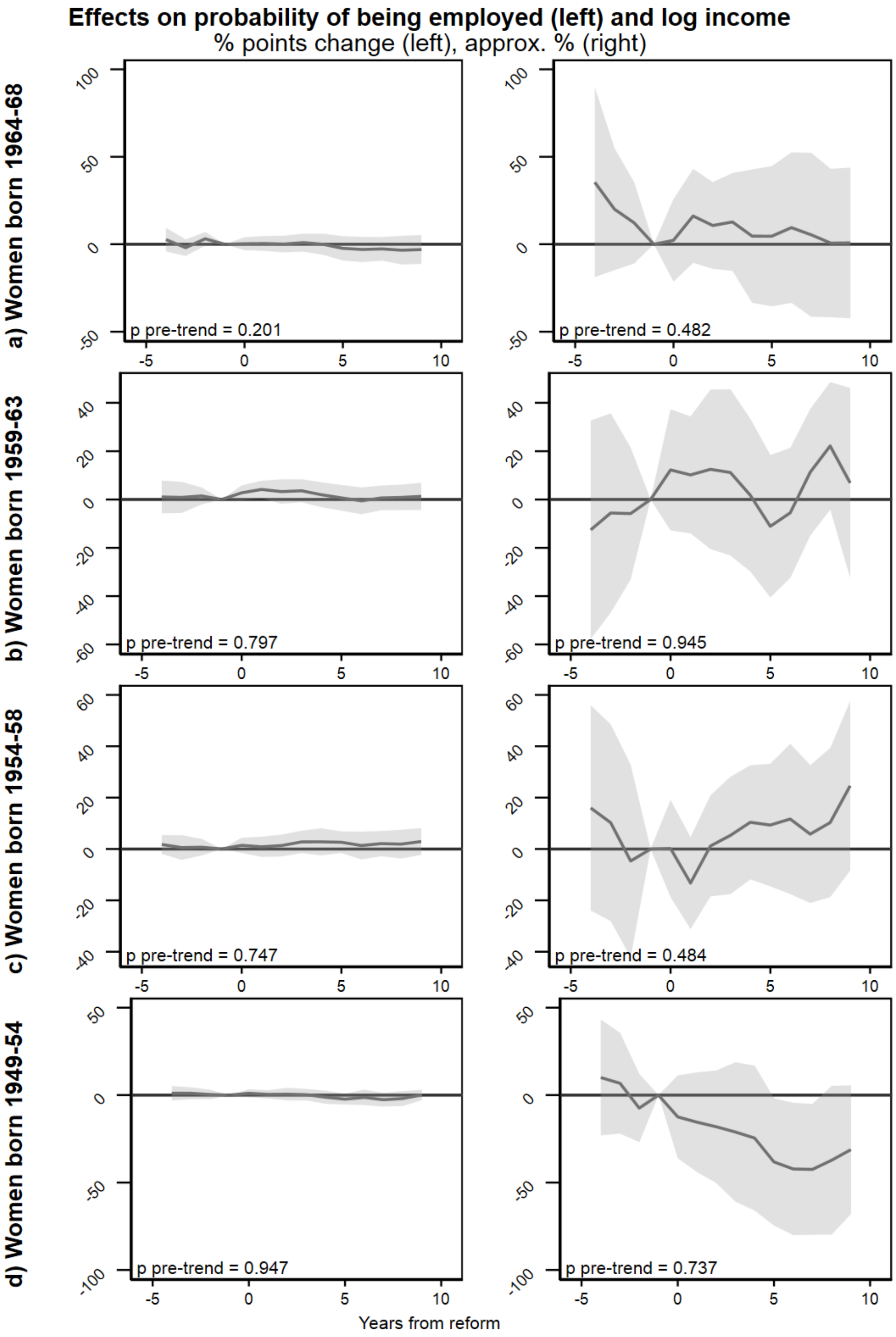

Note: Standard errors are clustered at the municipality level using the wild cluster bootstrap. P-values give the result of joint tests of the statistical significance of the estimates in the pre-period. Estimates are controlled for dummy variables for age and calendar time in years, dummy variables for municipality of residence in 1988.

Figure S.7: Event study estimates for the probability of being in education (left panel) and having completed higher education (right panel). Separate estimates by birth cohort.

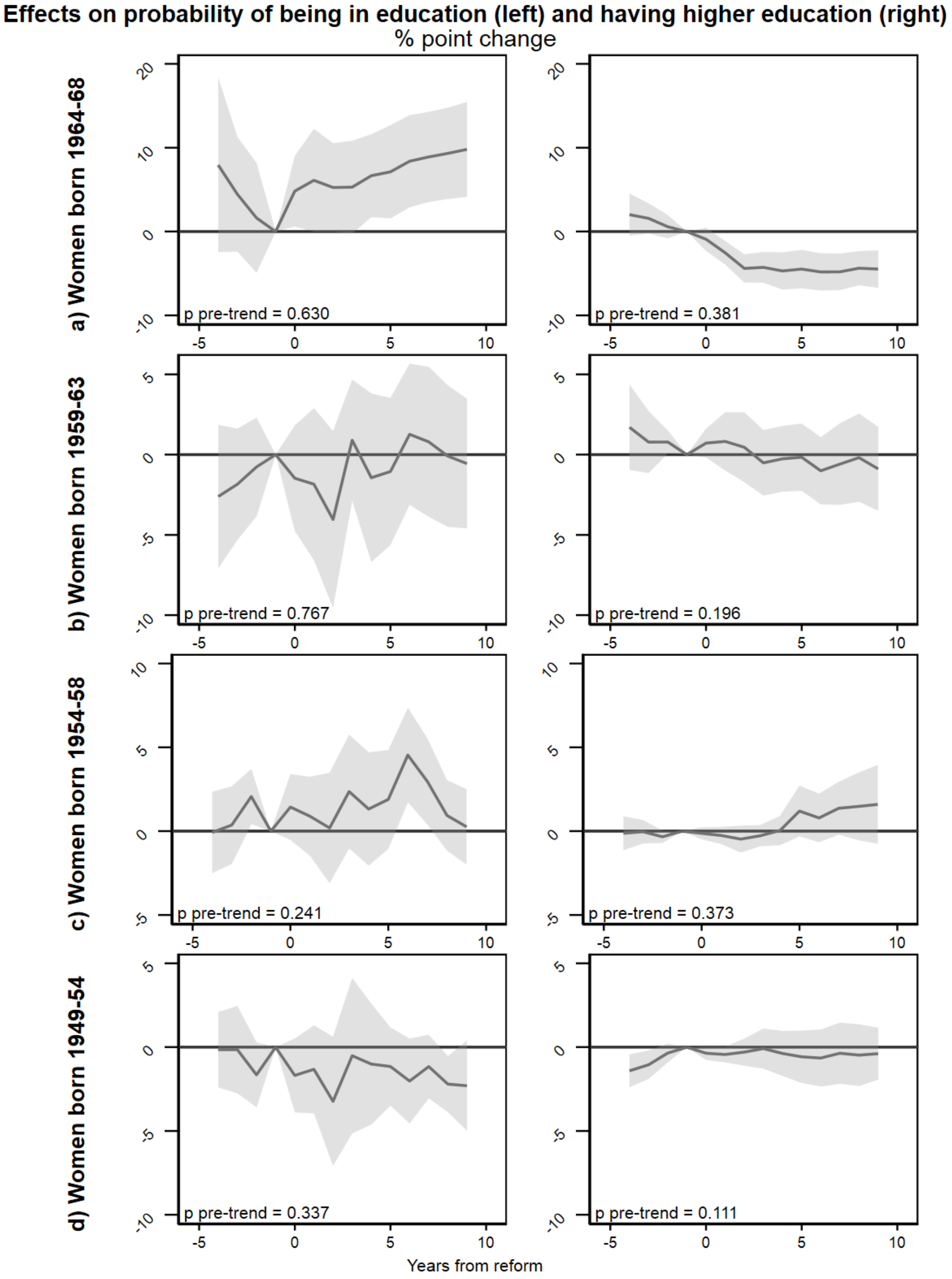

Note: Standard errors are clustered at the municipality level using the wild cluster bootstrap. P-values give the result of joint tests of the statistical significance of the estimates in the pre-period. Estimates are controlled for dummy variables for age and calendar time in years, dummy variables for municipality of residence in 1988.

Figure S.8: Event study estimates for effects of number of children, and the probability of having at least one child. Inverse probability weighing. Separate estimates by birth cohort.

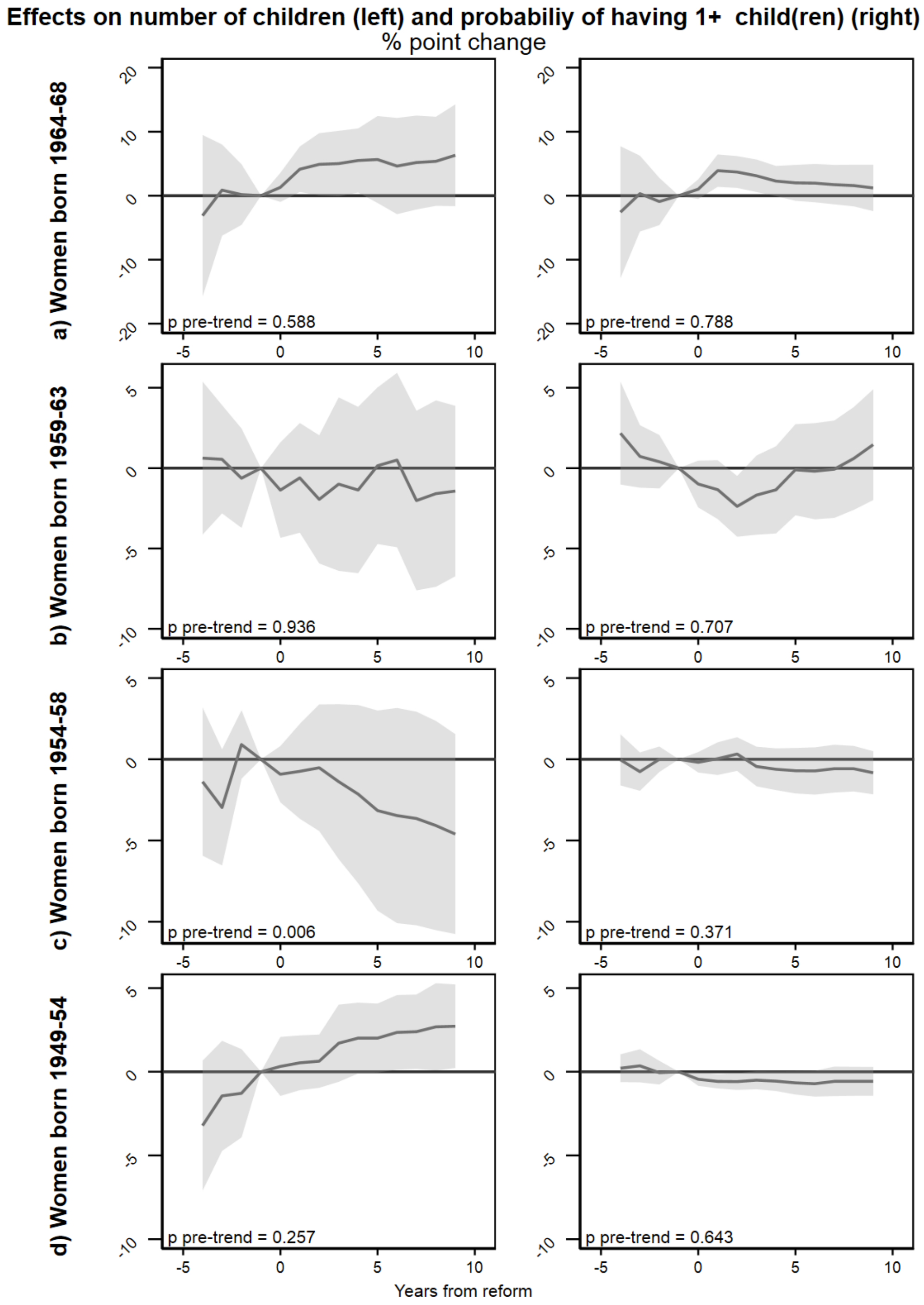

Note: Standard errors are clustered at the municipality level using the wild cluster bootstrap. P-values give the result of joint tests of the statistical significance of the estimates in the pre-period. Estimates are controlled for dummy variables for age and calendar time in years, dummy variables for municipality of residence in 1988. Inverse Probability Weighing includes covariates for age, marital status, educational attainment, educational enrollment, and age of youngest child, all measured in 1988.
